# An integrative review on treatment guidelines for complicated urinary tract infections: a synthesis of evidence-based recommendations

**DOI:** 10.1590/S1678-9946202567007

**Published:** 2025-02-07

**Authors:** Amanda Azevedo Bittencourt, Marina Della Negra de Paula, Ana Carolina Padula Ribeiro-Pereira, Paula de Mendonça Batista, Thales José Polis

**Affiliations:** 1 MSD, Global Medical Affairs, São Paulo, São Paulo, Brazil; 2 Origin Health, São Paulo, São Paulo, Brazil

**Keywords:** Urinary tract infections, Practice guideline, Literature review

## Abstract

Urinary tract infections (UTI) lie among the most common bacterial infections worldwide. Since their manifestations can range from laboratory findings (asymptomatic bacteriuria) to septic shock, using appropriate antimicrobial agents is crucial to avoid complications and the misuse of antibiotics. This study aims to review scientific publications and the main guidelines to treat complicated UTI. A literature review was carried out in September 2022 on the LILACS, MEDLINE via PubMed, and SciELO databases. Descriptors, keywords, and MeSH terms were used to develop search strategies. Full documentation meeting the following criteria was included: management of patients with a diagnosis of complicated UTI; guidelines, recommendations, consensus articles, expert opinion articles (with recommendations), and meta-analyses including data from randomized controlled trials; and articles published from 2001 to 2022. Articles published in languages other than English, Spanish, French, and Portuguese and those unrelated to complicated UTI were excluded. After applying the eligibility criteria, 28 studies were included in this review. Fluoroquinolones are the most frequently recommended option for complicated cystitis and pyelonephritis. Guideline recommendations for recurrent UTI include antibiotic prophylaxis and treatment. Guidelines developed to propose treatment strategies for the pediatric population typically stratify cases according to their infection site (upper or lower),and the presence of fever. Guidelines propose different approaches, likely related to local antibiotic resistance and varying clinical manifestations. In this context, antimicrobial stewardship practices are essential to promote the adequate use of antibiotics for complicated UTI and to avoid antimicrobial resistance.

## INTRODUCTION

Urinary tract infections (UTI) may constitute some of the most common bacterial infections requiring medical assistance worldwide^1^. Although its occurrence is usually related to mild symptoms, the inappropriate use of antibiotics can lead to resistance^2^. Thus, it is essential to establish the appropriate criteria to determine the treatment strategy with specific spectrum and optimal course of antibiotics^2^. Around 60% of all women have at least one symptomatic UTI episode during their lifetime, with the highest incidence estimate occurring in the youngest and most sexually active of them^2,3^. The administration of antibiotic therapy shows significant symptomatic and bacteriological cure rates and better prevention of reinfection, whereas about 25% of this population experiences spontaneous resolution of symptoms^2^. UTI prevalence among men is significantly lower than in women and occurs mainly among those with urologic structural abnormalities and older adults^2^. UTI commonly occurs in children, showing a higher incidence in girls aged older than one year and uncircumcised boys. By the age of 16 years, 11.3% of girls and 3.6% of boys will have had a UTI^4^.

Urine culture is the main tool to diagnose UTI. It provides information about the causative microorganism, enabling proper disease management^5^. Given the low sensitivity of urine culture to detect acute UTI, new methods such as next-generation sequencing and polymerase chain reaction have been proposed^6,7^. Szlachta-McGinn *et al*.^8^ showed, by a meta-analysis, that next-generation sequencing may be more sensitive in detecting urinary bacteria, highlighting the need for more evidence of the benefit of the molecular testing in clinical practice^6^. Moreover, complementary exams such as ultrasound and computerized tomography may exclude the possibility of the occurrence of obstructive pathologies.

UTI may be classified as upper (pyelonephritis) or lower (cystitis, prostatitis) and as uncomplicated or complicated according to the site of infection, underlying diseases, and anatomical or functional urinary tract abnormalities^9^. Complicated UTIs carry a higher risk of treatment failure and are usually associated with require longer courses of treatment and different antibiotics. Some examples of complicated UTI may include those in males due to anatomical abnormalities, immunocompromised states, atypical causative organisms, pregnancy, instrumentation, renal transplants, spinal cord injuries, impaired renal function, surgical prostatectomies or radiotherapy^10^. Considering that UTI manifestations may range from laboratory findings (asymptomatic bacteriuria) to septic shock, the appropriate understanding of these symptoms and use of appropriate antimicrobial agents are crucial to prevent serious complications and misuse of antibiotics, which reduce the expression of resistant bacteria^9^.

Antibiotic resistance of pathogens related to UTI has significantly increased, and healthcare providers have become aware of the risk of non-rational systematic antibiotic use. Several antibiotic agents exert a variety of selective pressures on the pathogens involved in the infection and on unrelated microbiota at that same site^11^. International guidelines on the management of uncomplicated and complicated UTI have been reassessed since then^2,11^.

We can understand the increase in antimicrobial resistance (AMR) in the last few years by several studies. In a study carried out from 2018 to 2020 in Latin America, 6.3% of all non-*Morganellaceae* Enterobacterales isolates carried a *Klebsiella pneumoniae* carbapenemase enzyme (metallo-β-lactamase-negative), 1.8% a metallo-β-lactamase, and 0.4% an OXA-48-like carbapenemase (metallo-β-lactamase-negative)^12^. In Brazil, a study carried out from 2016 to 2017 showed that the most common pathogens of urinary tract infections referred to *Escherichia coli* and *K. pneumoniae* ^13^. Ceftriaxone resistance in all *E. coli* isolates totaled 28.3% and 63.8% in *K. pneumoniae* isolates^13^. Resistance to ciprofloxacin among isolates of these two pathogens totaled 44.9% and 68.4%, respectively^13^. Resistance to carbapenems is even more evident among *K. pneumoniae* isolates, with 39.9% meropenem resistance in this last study^13^. Given the background and future therapeutic scenario of antimicrobials, this study aims to evaluate the scientific publications and main guidelines to treat complicated UTI. Its literature review also supports the proposal of an evidence-based treatment protocol grounded in the best evidence in the clinical literature.

## MATERIALS AND METHODS

### Study design

An integrative review was performed to answer the following research question: “Which guidelines and scientific evidence have been used as elements of care in the management of UTI to provide better assistance, create a greater perception of value in health, and offer the best journey for the patient?”.

### Literature search

A search was performed in September 2022 on the Latin American and Caribbean Health Sciences Literature, MEDLINE via PubMed, and Scientific Electronic Library Online databases considering articles that had been published from 2001 to 2022. To answer the guiding question, a selection of descriptors was carried out, according to the Health Sciences Descriptors and Medical Subject Headings. The controlled descriptors and Boolean operators (“And” and “OR”) were selected when necessary: “Urinary Tract Infections,” “Cystitis,” “Urethritis,” “Pyelonephritis,” “Therapeutic Guidelines,” “Metanalysis,” “Randomized Controlled Trial.” Inclusion criteria were based on language (only considering studies published in English, Spanish, French, and Portuguese), species (only humans), and methodological design (guidelines, recommendations, consensus, and structured reviews). Descriptors, keywords, and Medical Subject Headings terms according to the characteristics of each database were used to build search strategies, and the detailed information used for each database is shown in Supplementary Table S1.

### Study selection

In total, two reviewers analyzed and applied the eligibility criteria defined for this study. In case of disagreements, a third reviewer was consulted to provide a final decision. Full documentation meeting the following criteria was included in this review: involving the management of patients with a complicated UTI diagnosis including cystitis and pyelonephritis; guidelines, recommendations, consensus papers, expert opinion papers including recommendations, and meta-analyses including data from randomized controlled trials; and articles published from 2001 to 2022. Clinical trials at any phase, molecular biology studies, preclinical trials, narrative reviews (without recommendations), position papers, and observational studies were excluded. Articles published in languages other than English, Spanish, French, and Portuguese or unrelated to complicated UTIs were also excluded.

### Variables

Data from eligible documents were extracted by two reviewers and registered on an Excel^®^ spreadsheet. A third reviewer was consulted to provide a final decision when no consensus about issues related to data collection was achieved.

Variables related to study characteristics (study design, year of publication, country of publication, objectives), population (UTI in general or cystitis and/or pyelonephritis alone), and characteristics of the proposed treatment (therapeutic options, posology, order of choice, use at intensive care unit) were extracted from selected studies. The best available therapy information was extracted from meta-analyses including data from randomized controlled trials in order to determine treatment strategies used in different study sites.

### Assessment of study quality

The quality of the included studies was assessed using a validated instrument to identify possible biases and their impact on conclusions. The Appraisal of Guidelines for Research & Evaluation II was used since it is a generic instrument to address variability in the quality of published guidelines by evaluating methodological rigor and transparency during document preparation. It can be used to assess guidelines for any disease and at any stage of care, including aspects related to health promotion, screening, diagnosis, and treatment^14^. A Measurement Tool to Assess Systematic Reviews was used to assess the quality of systematic reviews. This instrument was developed to evaluate systematic reviews of randomized controlled trials^15^.

The Grading of Recommendations Assessment, Development and Evaluation was used to weigh aspects that may increase or decrease the quality of evidence on the effect of an outcome intervention. Moreover, two reviewers carried out this assessment, and divergences were resolved by a third party.

## RESULTS

### Search results

The search yielded 1,070 results, including duplicates, 1,069 from electronic databases, and one from a manual search. The eligibility criteria included 28 references in this review^9,16-42^. Supplementary Figure S1 shows the inclusion flowchart. Table 1 shows the characteristics of the included guidelines and the assessment of the evidence quality. Moreover, 12 publications were carried out in America; 11, in Europe; four, in Asia; and one, in Australia.

**Table 1:** General characteristics of the included guidelines.

Article	Country/Region	Population/Indication	Overall Quality – AGREE II
Tamma *et al.* ^18^ (Infectious Diseases Society of America)	America	Urinary tract infections in general	6/7
Wong *et al.* ^17^	Asia	Complicated UTIs with neurogenic bladder	4/7
Buettcher *et al.* ^37^	Switzerland	Urinary tract infections in children	6/7
Chuang *et al.* ^42^	Asia	Catheter-associated urinary tract infection	5/7
‘t Hoen *et al.* ^41^ (European Association of Urology and European Society for Paediatric Urology)	Europe	Urinary tract infections in children	6/7
Nemirovsky *et al.* ^23^	Argentina	Urinary tract infections in general	4/7
Nemirovsky *et al.* ^24^	Argentina	Urinary tract infections in general	4/7
Hevia *et al.* ^28^ (Chilean Society of Pediatrics)	Chile	Urinary tract infections in children	6/7
Pérez *et al.* ^21^ (Asociación Española de Pediatría)	Spain	Urinary tract infections in children	6/7
Brubaker *et al.* ^38^ (American Urogynecologic Society)	United States	Recurrent urinary tract infections in women	5/7
Kang *et al.* ^9^	South Korea	Community-acquired urinary tract infections	7/7
De Cueto *et al.* ^33^ (Spanish Society of Clinical Microbiology and Infectious Diseases)	Spain	Urinary tract infections in general	5/7
Epp *et al.* ^31^ (Society of Obstetricians and Gynecologists of Canada)	Canada	Recurrent urinary tract infections in women	5/7
Yamamoto *et al.* ^16^ (Japanese Association for Infectious Disease/Japanese Society of Chemotherapy)	Japan	Urinary tract infection among adults and male genital tract infections	6/7
Cohen *et al.* ^35^ (Pediatric Infectious Diseases Group of the French Pediatrics Society and the French-Language Infectious Diseases Society)	France	Urinary tract infections in children	6/7
McTaggart *et al.* ^26^	Australia	Urinary tract infections in children	5/7
Stein *et al.* ^19^ (European Association of Urology and European Society for Paediatric Urology)	Europe	Urinary tract infections in children	5/7
Dayts *et al.* ^34^	United States	Catheter-associated urinary tract infection within adult neurocritical care	4/7
Ammenti *et al.* ^40^	Italy	Febrile urinary tract infections in young children	5/7
Chishti *et al.* ^36^	United States	Inpatient care of children with pyelonephritis	5/7
Epp *et al.* ^30^ (Society of Obstetricians and Gynecologists of Canada)	Canada	Recurrent urinary tract infections in women	5/7
Hooton *et al.* ^27^	America	Catheter-associated urinary tract infection in adults	7/7
Velázquez *et al.* ^32^ (Mexican College of Specialists in Gynecology and Obstetrics)	Mexico	Recurrent urinary tract infections in women	6/7
French Agency for the Safety of Health Products^29^	France	Urinary tract infection among adults	6/7
Baumer *et al.* ^39^	United Kingdom	Urinary tract infections in children	5/7
Nicolle *et al.* ^22^	Canada	Complicated urinary tract infections among adults	6/7
Naber *et al.* ^25^ (European Association of Urology)	Europe	Urinary tract infection among adults and male genital tract infections	4/7
Riedmiller *et al.* ^20^ (European Association of Urology)	Europe	Urinary tract infections among children	4/7

This study used the Appraisal of Guidelines for Research and Evaluation II, a generic tool designed to assess the quality of clinical practice guidelines, showing the final score the reviewer attributed to each study. Most guidelines achieved high overall scores (≥70%) and received a classification as studies strongly recommended, showing solid methodological quality and practical relevance. On the other hand, six guidelines scored 50-69%, indicating moderate quality. This review classified these studies as recommended with adjustments, suggesting that they provide generally reliable recommendations but could benefit from improvements in clarity, methodological rigor, or practical applicability.

### Treatment for complicated cystitis

A total of three guidelines proposed recommendations for complicated cystitis. Nitrofurantoin is a therapeutic option proposed by one guideline, whereas other first-choice options included fluoroquinolones (levofloxacin, ciprofloxacin, tosufloxacin, sitafloxacin), aminopenicillin/β-lactamases inhibitors (amoxicillin/clavulanic acid, sultamicillin) or second or third generation cephalosporins and aminoglycosides. Table 2 shows such overall information.

**Table 2:** Guidelines including treatment recommendations for cystitis.

Article	General Recommendations
Yamamoto *et al.* ^16^ (Japanese Association for Infectious Disease/Japanese Society of Chemotherapy)	Complicated: First choice: oral levofloxacin, ciprofloxacin, or tosufloxacin or sitafloxacin or clavulanic acid/amoxicillin or sultamicillin; Alternatives: oral cefdinir or cefpodoxime proxetil or cefcapene pivoxil hydrochloride; Refractory cases: intravenous meropenem or doripenem or imipenem/cilastatin sodium or cefepime or cefozopran or tazobactam/piperacillin.
French Agency for the Safety of Health Products^29^	Complicated if treatment cannot be delayed: First-line: nitrofurantoin ≥seven days; Second-line: ≥5-day cefixime or ≥5-day fluoroquinolone. Complicated if treatment can be delayed: Antibiotic according to antibiogram results. Recurrent cystitis should be treated according to risk factor analysis (if related or not to sex).
Naber *et al.* ^25^ (European Association of Urology)	UTI with complicating factors: Recommended: fluoroquinolone or aminopenicillin/ ß-lactamase-inhibitor or 2^nd^ Gen. cephalosporin or 3a Gen. cephalosporin or aminoglycoside; In case of failure of initial therapy within 1–3 days or in clinically severe cases: fluoroquinolone, if not used initially, or acylaminopenicillin/ b-lactamase-inhibitor or 3b Gen. cephalosporin or carbapenem ± aminoglycoside; In cases of *Candida*: fluconazole or amphotericin B.

### Treatment for pyelonephritis

A total of six guidelines proposed recommendations for complicated pyelonephritis management. All guidelines proposed fluoroquinolone. Tamma *et al.* ^18^ stratifies treatment according to pathogens and recommends carbapenems, fluoroquinolones, or sulfonamides for individuals with extended-spectrum β-lactamase-producing Enterobacterales, fluoroquinolones or sulfonamides for patients with carbapenem-resistant Enterobacterales, and β-lactamases inhibitors and carbapenems for individuals with *Pseudomonas aeruginosa* with difficult-to-treat resistance. Table 3 shows such overall information.

**Table 3:** Guidelines including treatment recommendations for pyelonephritis.

Article	General Recommendations
Tamma *et al*.^18^ (Infectious Diseases Society of America)	Pyelonephritis and UTIs, according to pathogen: Extended-spectrum β-lactamase-producing Enterobacterales: ertapenem, meropenem, imipenem/cilastatin, ciprofloxacin, levofloxacin, or trimethoprim-sulfamethoxazole; Carbapenem-resistant Enterobacterales: ciprofloxacin, levofloxacin, or trimethoprim-sulfamethoxazole; *Pseudomonas aeruginosa* with difficult-to-treat resistance: ceftolozane/tazobactam, ceftazidime/avibactam, imipenem/cilastatin/relebactam, or cefiderocol.
Nemirovsky *et al*.^24^	First-choice: ciprofloxacin; Second choice: ceftriaxone; Severe symptoms, hemodynamic instability or digestive intolerance: hospitalization and the start of parenteral treatment with ceftriaxone, cefazolin, or amikacin; Nitrofurantoin and fosfomycin are not recommended.
Kang *et al.* ^9^	Complicated pyelonephritis related to urinary tract obstruction: Follow the protocol for uncomplicated disease, except if clinical symptoms are severe (antibiotic selection should be based on the treatment protocol for severe UTIs accompanied by sepsis); Possible antibiotics: fluoroquinolone, β-lactam/β-lactamase inhibitor, broad-spectrum cephalosporin, aminoglycoside, or carbapenem; Pyelonephritis accompanied by sepsis or for recurrent pyelonephritis: piperacillin/tazobactam, broad-spectrum third-generation or fourth-generation cephalosporins, and carbapenem.
Yamamoto *et al.* ^16^ (Japanese Association for Infectious Disease/Japanese Society of Chemotherapy)	Complicated – mild/moderate conditions: First-choice: oral levofloxacin or ciprofloxacin or tosufloxacin or sitafloxacin; Alternatives: oral cefditoren pivoxil, cefpodoxime proxetil, or pivampicillin and cefcapene pivoxil hydrochloride. Complicated – severe conditions: First-choice: intravenous ceftazidime or ceftriaxone or tazobactam/piperacillin; Alternatives: intramuscular or intravenous amikacin, pazufloxacin, levofloxacin, cefepime, or imipenem/cilastatin sodium, meropenem, or doripenem.
French Agency for the Safety of Health Products^29^	Acute complicated: Probabilistic: injectable ceftriaxone or cefotaxime or subcutaneous fluoroquinolone; Oral route (after antibiogram results): amoxicillin or amoxicillin-clavulanic acid, cefixime fluoroquinolone, or sulfamethoxazole-trimethoprim for 10-14 days (sometimes, > 21 days).
Naber *et al.* ^25^ (European Association of Urology)	UTI with complicating factors: Recommended: fluoroquinolone, aminopenicillin/ß-lactamase-inhibitor, 2^nd^ Gen. cephalosporin, 3^rd^ Gen. cephalosporin, or aminoglycoside; In case of failure of initial therapy within 1–3 days or in clinically severe cases: fluoroquinolone if not used initially or acylaminopenicillin/ß-lactamase-inhibitor, 3b Gen. cephalosporin, carbapenem ± aminoglycoside; In cases of *Candida*: fluconazole or amphotericin B.

### Treatment for recurrent urinary tract infection

A total of four guidelines proposed recommendations for managing recurrent urinary tract infections. Infection should be managed by nitrofurantoin, trimethoprim/sulfamethoxazole, fosfomycin trometamol, or quinolones as a first-line treatment based on to the antibiogram result. Regarding prophylactic treatment, documents agree that it should only be initiated after a negative culture and that nitrofurantoin is an option. Table 4 shows such overall information.

**Table 4:** Guidelines including treatment recommendations for recurrent urinary tract infections.

Article	General Recommendations
Brubaker *et al.* ^38^ (American Urogynecologic Society)	Antibiotic treatment First-line: nitrofurantoin monohydrate/macrocrystals, trimethoprim/sulfamethoxazole, or fosfomycin trometamol; Second-line: fluoroquinolones or β-Lactams.
Epp *et al*.^31^ (Society of Obstetricians and Gynecologists of Canada)	Antibiotic prophylaxis Should not be initiated until a negative culture (carried out one to two weeks following treatment) has confirmed eradication of the infection; Continuous daily antibiotic prophylaxis for six to 12 months to women who had ≥ two urinary tract infections in six months or ≥three urinary tract infections in 12 months: cotrimoxazole, nitrofurantoin, cephalexin, trimethoprim, trimethoprim-sulfamethoxazole, or a quinolone.
Epp *et al.* ^30^ (Society of Obstetricians and Gynecologists of Canada)	Antibiotic prophylaxis Should not be initiated until a negative culture (conducted one to two weeks following treatment) has confirmed eradication of the infection. Continuous daily antibiotic prophylaxis for six to 12 months to women who had ≥ two urinary tract infections in six months or ≥ three urinary tract infections in 12 months: cotrimoxazole, nitrofurantoin, cephalexin, trimethoprim, trimethoprim-sulfamethoxazole, or a quinolone.
Velázquez *et al*.^32^ (Mexican College of Specialists in Gynecology and Obstetrics)	Antibiotic treatment Recommended: quinolones (ciprofloxacin) or nitrofurantoin or fosfomycin; Symptomatic uncomplicated episodes of urinary tract infection confirmed by urinalysis should be treated with antibiotics according to local sensitivity and resistance. Antibiotic prophylaxis Recommended in patients with unidentified causes or that are being studied; Recommended: daily nitrofurantoin.

### Treatment for catheter-associated urinary tract infection

A total of six guidelines proposed recommendations for catheter-associated urinary tract infection. Most referred to indwelling urethral catheters and recommend urine culture before initiating antimicrobial therapy. Moreover, discontinuation or catheter exchange is recommended. Table 5 shows such overall information.

**Table 5:** Guidelines including treatment recommendations for catheter-associated urinary tract infection.

Article	General Recommendations
Chuang *et al.* ^42^	Authors state that no single set of recommendation for empiric antibiotics could be made and provide the following suggestions: Monitoring uropathogens and local antibiotic resistance patterns; Urine cultures, preferably before initiation of antibiotic therapy, to guide the choice of definitive antibiotic therapy; Empiric antimicrobial therapy could be guided by recent prior urine culture results where possible; Early de-escalation of antibiotic therapy, guided by urine culture results, to the narrowest spectrum antibiotic available; Shorter five-day course of antibiotics with catheter exchange may be considered in treating catheter-associated urinary tract infection in patients with spinal cord injury; Asymptomatic bacteriuria should not be routinely treated with antibiotics.
Nemirovsky *et al.* ^24^	Antibiotic treatment — epidemiological factors such as prevalent germs, resistance patterns in different units, and regional prevalence, and individual factors should be considered for the choice of antimicrobial: Patients without risk factors: third-generation cephalosporins, piperacillin/tazobactam, or aminoglycosides; In case of suspicion of extended-spectrum β-lactamase-producing *Enterobacterales* (ESBL): aminoglycosides or carbapenems; In patients colonized by carbapenemase-producing *Enterobacterales* (CPE): colistin, fosfomycin, or amikacin.
De Cueto *et al.* ^33^ (Spanish Society of Clinical Microbiology and Infectious Diseases)	Antibiotic treatment - indicated for patients with symptomatic infection or clinical signs of sepsis: Parenteral broad-spectrum antibiotics adapted to the local resistance patterns of uropathogens — imipenem, meropenem, and piperacillin/tazobactam; If the patient has septic shock or resistance to β-lactams is suspected, combination therapy with amikacin should be considered.
Yamamoto *et al*.^16^ (Japanese Association for Infectious Disease/Japanese Society of Chemotherapy)	Antibiotic treatment — should be selected in consideration of the susceptibility pattern of gram-negative rods at each institution: First-choice: intravenous piperacillin/tazobactam or ceftazidime or cefepime or meropenem; Alternatives: intravenous ciprofloxacin, intramuscular or intravenous gentamicin, intramuscular or intravenous amikacin, intravenous pazufloxacin, or intravenous levofloxacin.
Dayts *et al.* ^34^	General recommendations: Urine culture before initiating antimicrobial therapy; Catheter replacement if still indicated and in place for >two weeks; Empiric antimicrobial treatment. Antibiotic treatment: Seven-day regimen for patients with prompt resolution of symptoms and 10–14 days of treatment are recommended for those with a delayed response, regardless of whether a patient remains catheterized; Five-day regimen of levofloxacin could be considered in patients with catheter-associated urinary tract infection who have no severe illnesses; Three-day regimen could be considered for women ages ≤65 years without upper urinary tract symptoms after an indwelling catheter has been removed; 14-day regimen is recommended for patients with spinal cord injury.
Hooton *et al.* ^27^	General recommendations: Urine culture before initiating antimicrobial therapy. Antibiotic treatment: Seven -day regimen for patients with prompt resolution and 10-14 days for those with delayed response; Five-day regimen of levofloxacin could be considered in patients with catheter-associated urinary tract infection who are not severely ill; Three -day regimen could be considered for women ages ≤65 years without upper urinary tract symptoms after an indwelling catheter has been removed.

### Treatment for complicated urinary tract infections among children

A total of 11 guidelines proposed recommendations for managing UTIs among children. Moreover, two guidelines recommended the treatment of febrile UTI with a parenteral broad-spectrum antibiotic or intravenous amoxicillin plus aminoglycoside, whereas one only suggested intravenous treatment if fever is associated with other criteria and when oral treatment is impossible. Antimicrobial agents are recommended according to patients’ age and route of administration. Penicillins, cephalosporins, sulphonamides, fluoroquinolones, nitrofurantoin, and aminoglycosides may be considered, as in Table 6.

**Table 6:** Guidelines including treatment recommendations for urinary tract infections among children.

Article	General recommendations
Buettcher *et al.* ^37^	Fever, pyelonephritis Choice: intravenous amoxicillin + aminoglycoside; Alternative: intravenous amoxicillin + ceftriaxone, amoxicillin-clavulanate, or 3^rd^ Gen. cephalosporine.
‘t Hoen *et al.* ^41^ (European Association of Urology and European Society for Paediatric Urology)	Parenteral therapy; Febrile UTIs: treatment for 4-7 day courses of parenteral therapy with broad-spectrum antibiotics.
Hevia *et al.* ^28^ (Chilean Society of Pediatrics)	Acute pyelonephritis Children with bacteriuria plus fever ≥38 °C: oral antibiotic treatment for 7-10 days; Children with bacteriuria, fever <38 °C and back pain or fist percussion + reliable urine test. Urine culture if not done: if oral treatment is impossible, consider initial IV treatment for one to three days and then continue with oral antibiotic.
Pérez *et al.* ^21^ (Asociación Española de Pediatría)	Acute pyelonephritis Outpatient management: 7-10 days of cefixime or ceftibuten; Child hospitalized < three months: ampicillin + gentamicin or ampicillin + cefotaxime; Child hospitalized > three months: gentamicin or cefotaxime or ceftriaxone.
Cohen *et al.* ^35^ (Pediatric Infectious Diseases Group of the French Pediatrics Society and the French-Language Infectious Diseases Society)	Pyelonephritis and febrile UTI in infants and young children (two to three days on average until the antibiogram result, then relay depending on the antibiogram for an average total duration of 10 days). Child hospitalized (<three months or sepsis, or known severe underlying uropathy): intravenous cefotaxime or ceftriaxone plus amikacin; Child over three months in the pediatric emergency room with no need for hospitalization, if intravenous therapy: amikacin or ceftriaxone; Child over three months in the pediatric emergency room with no need for hospitalization, if intramuscular therapy: ceftriaxone; Child over three months in the pediatric emergency room with no need for hospitalization, if oral therapy: cefixime.
McTaggart *et al.* ^26^	Acute pyelonephritis In children older than one month of life with acute pyelonephritis: oral treatment if low risk of serious illness, shows no sepsis, and can tolerate oral medications; Single-dose treatment is not recommended.
Stein *et al*.^19^ (European Association of Urology and European Society for Paediatric Urology)	Pyelonephritis Pyelonephritis during the first 0–6 months of life: ceftazidime + ampicillin or aminoglycoside + ampicillin; Complicated pyelonephritis (with dilatation/reflux; severe bladder dysfunction) and/or urosepsis (all ages): ceftazidime + ampicillin or aminoglycoside + ampicillin.
Ammenti *et al.* ^40^	Treat parenterally and continue with an oral antibiotic after 2–4 days. Oral antibiotic therapy Suggested options: three-day amoxicillin-clavulanic acid or cefixime or ceftibuten. Intravenous antibiotic therapy Suggested alternatives: amoxicillin-clavulanic acid, ampicillin-sulbactam, cefotaxime, ceftriaxone, or aminoglycosides.
Chishti *et al.* ^36^	Pyelonephritis. Oral antibiotic therapy Penicillins: amoxicillin or augmentin; Cephalosporins: cephalexin or cefadroxil or cefuroxime or cefixime; Sulphonamides: trimethoprim-sulphamethoxazole; Fluoroquinolones: ciprofloxacilin; Nitrofurantoin. Intravenous antibiotic therapy Penicilins: ampicillin or ticarciclin; Aminoglycosides: gentamicin or amikacin; Cephalosporins: cefazolin or cefotaxime or ceftriaxone or ceftazidime or cefepime; Fluoroquinolones: ciprofloxacilin.
Baumer *et al.* ^39^	Infants younger than three months of age with a possible UTI should be referred immediately to the care of a pediatric specialist. Infants and children aged three months or older with acute pyelonephritis/UTI Treat with oral antibiotics for 7–10 days: oral antibiotic with low resistance patterns, for example cephalosporin or co-amoxiclav; If oral antibiotics cannot be used: intravenous antibiotic agent such as cefotaxime or ceftriaxone for 2–4 days followed by oral antibiotics for 10 days.
Riedmiller *et al.* ^20^ (European Association of Urology)	Pyelonephritis Intravenous antibiotic therapy with broad-spectrum penicillin or cephalosporin.

### Compiled evidence on complicated urinary tract infections

Evidence on UTI has been compiled to aggregate information obtained by this literature review. Supplementary Figure S2 shows the flowchart with the first therapeutic options proposed for each assessed condition considering two different time periods.

## DISCUSSION

UTI constitute a significant public health concern due to their high prevalence and possible misuse of antibiotics, which can contribute to the development of AMR^1,2^. The World Health Organization implicated bacterial AMR in about five million deaths in 2019, underscoring its critical impact on global health^43^. Majigo *et al.* ^44^ proposed a surveillance protocol for AMR in low- and middle-income countries, using UTIs as a proxy for community-acquired infections. This approach aims to establish a sustainable surveillance system to monitor AMR. Therefore, understanding how clinical guidelines recommend managing UTIs across regions is crucial to inform evidence-based clinical decisions and promote optimal patient outcomes.

This review found 28 guidelines to treat complicated UTIs. They made it possible to evaluate the antibiotics treatment evolution over more than 20 years and determine the best options available to clinical practice. This study built an evidence-based care bundle to highlight all the recommended therapeutic options in 2001-2011 and 2012-2022. This context adds important knowledge to address UTI in clinical practice and to avoid AMR.

According to the three guidelines for cystitis in this review, first-line treatment should consider fluoroquinolones, nitrofurantoin, aminopenicillin/β-lactamase-inhibitor, second or third generation cephalosporin, or an aminoglycoside^16,25,29^. Yamamoto *et al.* ^16^ proposes the following option to manage complicated cystitis: oral levofloxacin, ciprofloxacin, tosufloxacin, sitafloxacin, clavulanic acid/amoxicillin, or sultamicillin. Guidelines should be carefully considered since they may represent the local context of the publication. For example, some of these drugs may be currently unavailable in several countries, especially tosufloxacin, sitafloxacin, and sultamicillin. Local medical societies should join efforts to develop guidelines that reflect both the local resistance profile and the reality of approved drugs of each country.

Fluoroquinolones configure the choice for complicated pyelonephritis^9,16,18,24,25,29^. In total, two guidelines proposed disease management considering sepsis and pathogen^9,18^. UTI preceding sepsis involve the recommended piperacillin/tazobactam, broad-spectrum third- or fourth-generation cephalosporins, and carbapenem^9^. Treatment with pathogen, carbapenems, fluoroquinolones, or sulfonamides are recommended for individuals with extended-spectrum β-lactamase-producing Enterobacterales and that with fluoroquinolones or sulfonamides for patients with carbapenem-resistant Enterobacterales and β-lactamases inhibitors and carbapenems for individuals with *P. aeruginosa* with difficult-to-treat resistance^18^.The Infectious Diseases Society of America proposed recommendations for infections from extended-spectrum β-lactamase producing Enterobacterales, carbapenem-resistant Enterobacterales, and *P. aeruginosa* with difficult-to-treat resistance as a strategy to address AMR in 2022. Ertapenem, meropenem, imipenem/cilastatin, ciprofloxacin, levofloxacin, or trimethoprim-sulfamethoxazole are considered the preferred treatment options for complicated UTIs from extended-spectrum β-lactamase producing Enterobacterales, whereas ciprofloxacin, levofloxacin, and trimethoprim-sulfamethoxazole configure the preferred treatment options for complicated UTI due to carbapenem-resistant Enterobacterales in cases of susceptibility. Ceftazidime/avibactam and imipenem/cilastatin/relebactam also constitute the preferred treatment options for complicated UTI due to carbapenem-resistant Enterobacterales. The Infectious Diseases Society of America guidance brought attention to the increased mortality and excess nephrotoxicity associated with polymyxin or aminoglycoside-based regimens regarding newer β-lactam-β-lactamase inhibitor agents to treat carbapenem-resistant Enterobacterales infections. Ceftolozane/tazobactam, ceftazidime/avibactam, and imipenem/cilastatin/relebactam configure the preferred treatment options for complicated UTI due to *P. aeruginosa* with difficult-to-treat resistance^18^. Although piperacillin/tazobactam has shown *in vitro* activity against extended-spectrum β-lactamase producing Enterobacterales, observational studies have obtained conflicting results regarding its effectiveness in treating infections due to ESBL-producing bacteria^18^. A clinical trial of bloodstream infections due to ceftriaxone-resistant *E. coli* or *K. pneumoniae* indicated inferior results with piperacillin/tazobactam than those for carbapenem therapy^45^.

Ertapenem has recently been compared with class 2 carbapenems (meropenem, imipenem, and biapenem) in the empirical treatment of bacteremia due to *Enterobacterales* resistant to third-generation cephalosporins. Ertapenem was considered non-inferior to other carbapenems in this setting^46^.

Guideline recommendations for recurrent UTI encompass antibiotic prophylaxis and treatment. Most agree that prophylactic antibiotics prescription to decrease the risk of future episodes may follow the discussion of risks, benefits, and alternatives^30-32^. Recurrent UTI treatment is usually based on nitrofurantoin, trimethoprim-sulfamethoxazole, or fosfomycin trometamol^30,32,38^. This review focused on drug treatment but some other measures may be applied. Brubaker *et al.* ^38^ reported that behaviors such as wiping away from the urethra, voiding before and after intercourse, increasing frequency of voiding, wearing certain types of underwear, avoiding douching, or avoiding hot tubs, bubble bath, or tampons are yet to be stablished as risk factors, highlighting that physicians should consider the contribution of gross fecal soilage (as in women with fecal incontinence). Recommendations for managing catheter-associated UTI involve considering local resistance patterns before treatment definition^16,24,27,34,42^. Regimens considering third-generation cephalosporins, levofloxacin, piperacillin/tazobactam, aminoglycosides, or carbapenems are the most frequently recommended^16,24,27,34^.

Guidelines to propose treatment strategies for the pediatric population usually stratify cases according to the infection site, such as upper or lower infection and fever^19,21,35,37,40,47^. Amoxicillin, cefotaxime and ceftriaxone are the most recommended strategies for pyelonephritis^19,21,35,37,39,40,47^.

This review has several limitations that warrant consideration. Its extensive literature review found no guidelines from certain regions, potentially limiting the global representativeness of its findings. Additionally, this review included only documents published within predefined databases, which may have excluded recommendations by local ministries of health and medical societies. Finally, the implementation of real-world studies evaluating the application of these guidelines in clinical practice is crucial to assess their impact on disease management and the incidence of AMR.

## CONCLUSION

This study broadly overviewed the treatment proposed worldwide for complicated UTIs considering several conditions for over 20 years. Several guidelines propose different approaches that are probably related to local antibiotic resistance and clinical manifestations.

In this context, antimicrobial stewardship practices are essential to promote the adequate use of antibiotics for complicated UTIs. We should handle this infection more carefully to avoid AMR and assertively treat it to prevent negative outcomes for patients, especially in older adults.
