# Supplementary material for: An integrative review on treatment guidelines for complicated urinary tract infections: a synthesis of evidence-based recommendations
Source: Rev Inst Med Trop Sao Paulo. 2025 Feb 7;67:e7. doi: 10.1590/S1678-9946202567007 (PMC11808710; doi:10.1590/S1678-9946202567007)
Supplement: Supplementary file 1 [file 1678-9946-rimtsp-67-S1678-9946202567007-suppl01.pdf]

REVISTA DO INSTITUTO DE MEDICINA TROPICAL DE SÃO PAULO

JOURNAL OF THE SÃO PAULO INSTITUTE OF TROPICAL MEDICINE

SUPPLEMENTARY MATERIAL

https://doi.org/10.48331/scielodata.AKHUWX

An integrative review on treatment guidelines for complicated urinary tract infections: a synthesis of evidence-based recommendations

Amanda Azevedo Bittencourt[1], Marina Della Negra de Paula[1], Ana Carolina Padula Ribeiro-Pereira[2], Paula de Mendonça Batista[1], Thales José Polis[1]

[1]MSD, Global Medical Affairs, São Paulo, São Paulo, Brazil

[2]Origin Health, São Paulo, São Paulo, Brazil

Correspondence to: Amanda Azevedo Bittencourt
MSD, Global Medical Affairs, Avenida Chucri Zaidan, 296, 11º andar, Edif. Torre Z, Vila Cordeiro, CEP 04583-110, São Paulo, SP, Brazil
Tel: +55 11 94272-2643

E-mail: amanda.bittencourt@merck.com

Received: 6 June 2024

Accepted: 6 December 2024

Supplementary Table S1 - Terms used in search strategies

Database	Search strategy
MEDLINE via PubMed	("Urinary Tract Infections"[Mesh] OR "Urinary Tract Infections" OR "Infection, Urinary Tract" OR "Infections, Urinary Tract" OR "Tract Infection, Urinary" OR "Tract Infections, Urinary" OR "Urinary Tract Infection" OR "Cystitis"[Mesh] OR "Cystitis" OR "Urethritis"[Mesh] OR "Urethritis" OR "Pyelonephritis"[Mesh] OR "Pyelonephritis" OR "Pyelonephritides" OR "Pyelonephritis, Acute Necrotizing" OR "Necrotizing Pyelonephritis") AND ("Practice Guideline"[Publication Type] OR "Practice Guideline" OR "Clinical Practice Guideline" OR "Clinical Guidelines" OR "Guideline"[Publication Type] OR "Guideline" OR ("Meta-Analysis" [Publication Type] AND "Randomized Controlled Trial" [Publication Type]))
LILACS	("Infecções Urinárias" OR "Urinary Tract Infections" OR "Infecciones Urinarias" OR "Infections urinaires" OR "Infecções do Sistema Urinário" OR "Cistite" OR "Cystitis" OR "Cistitis" OR "Cystite" OR "Uretrite" OR "Urethritis" OR "Uretritis" OR "Urétrite" OR "Uretrites" OR "Pielonefrite" OR "Pyelonephritis" OR "Pielonefritis" OR "Pyélonéphrite" OR "Pielonefrite Aguda Necrosante" OR "Pielonefrite Necrosante") AND ("Guia de Prática Clínica" OR "Practice Guideline" OR "Guía de Práctica Clínica" OR "Guide de bonnes pratiques" OR "Diretiva Prática" OR "Diretiva de Prática Clínica" OR "Diretiva de Prática Médica" OR "Diretiva para a Prática Clínica" OR "Diretiva para a Prática Médica" OR "Diretriz Prática" OR "Diretriz de Prática Clínica" OR "Diretriz de Prática Médica" OR "Diretriz para a Prática Clínica" OR "Diretriz para a Prática Médica" OR "Diretrizes Clínicas" OR "Guia de Boas Práticas" OR "Guia de Prática Médica" OR "Guia para a Prática Médica" OR "Recomendação de Boas Práticas" OR (("Metanálise" OR "Meta-Analysis" OR "Metaanálisis" OR "Méta-analyse" OR "Metanálises") AND ("Ensaio Clínico Controlado Aleatório" OR "Ensaio Clínico Controlado Randomizado" OR "Ensaio Controlado Aleatório" OR "Randomized Controlled Trial" OR "Ensayo Clínico Controlado Aleatorio" OR "Essai contrôlé randomisé")))

This is an open-access article distributed under the terms of the Creative Commons Attribution License.

Supplementary Table S1 - Terms used in search strategies (cont.)

SciELO	("Infecções Urinárias" OR "Urinary Tract Infections" OR "Infecciones Urinarias" OR "Infections urinaires" OR "Infecções do Sistema Urinário" OR "Cistite" OR "Cystitis" OR "Cistitis" OR "Cystite" OR "Uretrite" OR "Urethritis" OR "Uretritis" OR "Urétrite" OR "Uretrites" OR "Pielonefrite" OR "Pyelonephritis" OR "Pielonefritis" OR "Pyélonéphrite" OR "Pielonefrite Aguda Necrosante" OR "Pielonefrite Necrosante") AND ("Guia de Prática Clínica" OR "Practice Guideline" OR "Guía de Práctica Clínica" OR "Guide de bonnes pratiques" OR "Diretiva Prática" OR "Diretiva de Prática Clínica" OR "Diretiva de Prática Médica" OR "Diretiva para a Prática Clínica" OR "Diretiva para a Prática Médica" OR "Diretriz Prática" OR "Diretriz de Prática Clínica" OR "Diretriz de Prática Médica" OR "Diretriz para a Prática Clínica" OR "Diretriz para a Prática Médica" OR "Diretrizes Clínicas" OR "Guia de Boas Práticas" OR "Guia de Prática Médica" OR "Guia para a Prática Médica" OR "Recomendação de Boas Práticas" OR (("Metanálise" OR "Meta-Analysis" OR "Metaanálisis" OR "Méta-analyse" OR "Metanálises") AND ("Ensaio Clínico Controlado Aleatório" OR "Ensaio Clínico Controlado Randomizado" OR "Ensaio Controlado Aleatório" OR "Randomized Controlled Trial" OR "Ensayo Clínico Controlado Aleatorio" OR "Essai contrôlé randomisé")))

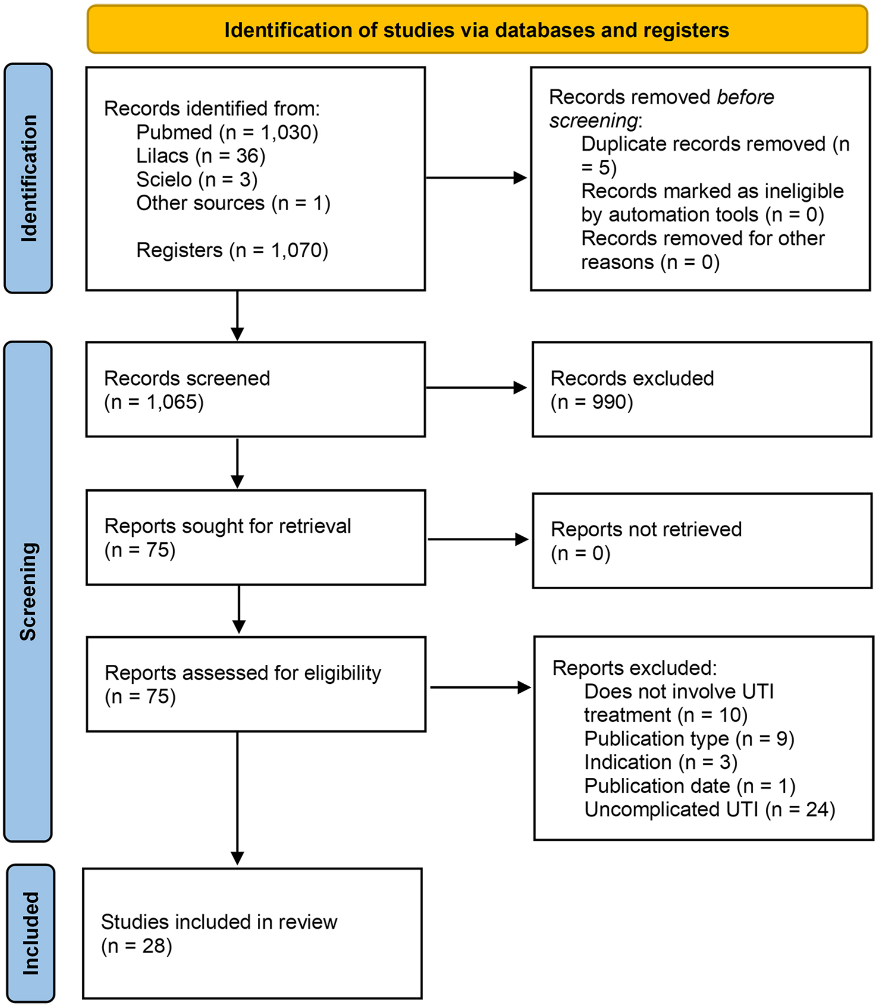

Supplementary Figure S1 - Study inclusion flowchart.

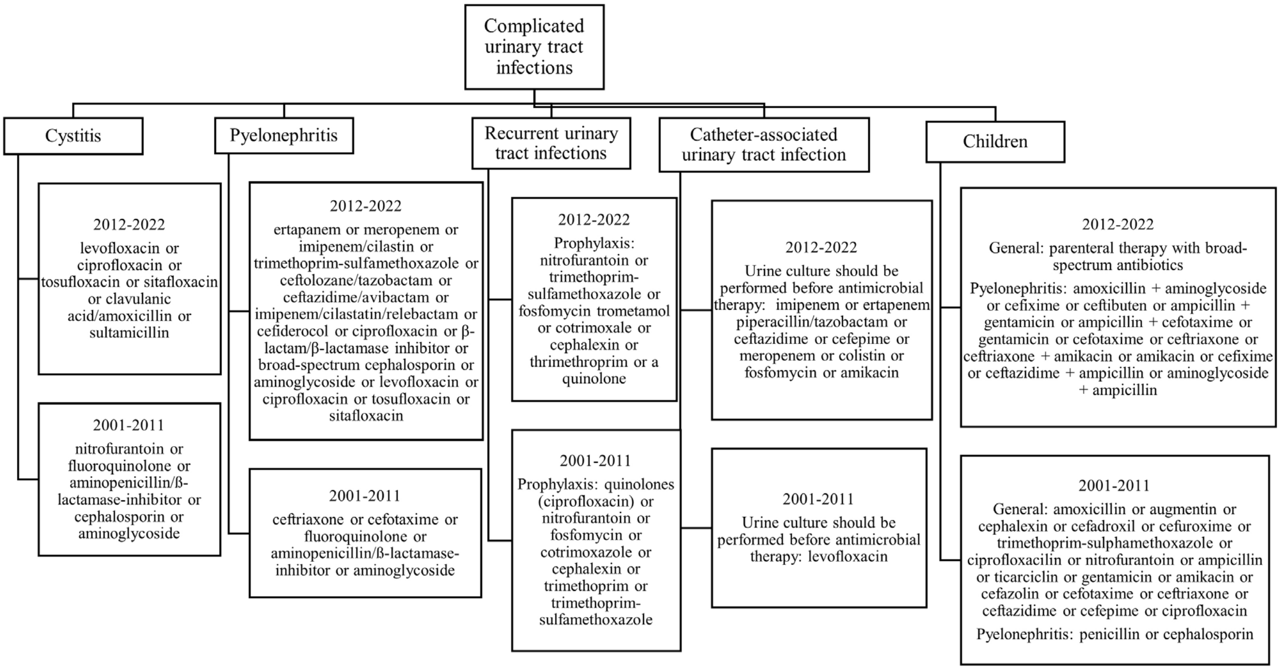

Supplementary Figure S2 - Compiled evidence on complicated urinary tract infections.
